# Nutrition gap in children, urban-rural: the key education and food. Colombia, 2015

**DOI:** 10.11606/s1518-8787.2020054001925

**Published:** 2020-11-04

**Authors:** Jhael N. Bermúdez, Daniel Ayala, Oscar F. Herrán

**Affiliations:** I Instituto Colombiano de Bienestar Familiar BogotáD.C Colombia Instituto Colombiano de Bienestar Familiar. Subdirección de Monitoreo y Evaluación. Bogotá D.C, Colombia; II Pontificia Universidad Católica de Chile Santiago Chile Pontificia Universidad Católica de Chile. Santiago, Chile; III Universidad Industrial de Santander Escuela de Nutrición y Dietética Bucaramanga Colombia Universidad Industrial de Santander. Escuela de Nutrición y Dietética. Bucaramanga, Colombia

**Keywords:** Child, Preschool, Nutrition Surveys, Food and Nutrition Security, Health Status Disparities, Rural Areas, Urban Area

## Abstract

**OBJECTIVE:**

To analyze the nutritional situation of children under five years old from both urban and rural areas of Colombia.

**METHOD:**

Analytical study, based on cross-sectional data, collected from ENSIN-2015. The sample consisted of 12,256 children aged between 0 and 4 years old. We calculated the prevalence ratios (PR) with their respective 95% confidence interval (95%CI). PR were assessed by binomial regression models with malnutrition or overweight as the dependent variable and geographic area as the explanatory variable. We used context variables to adjust the estimated PR and control the confounder within.

**RESULTS:**

Acute malnutrition (weight-for-height) had a prevalence of 1.6%, while overweight had a 5.6% rate. No differences per geographic zone in the weight-for-height indicator were found. Stunted growth – chronic malnutrition – was higher in the rural area (PR = 1.2; 95%CI 1–1.53; p = 0.050). Prevalences adjusted by variables related to structural, social and economic developement showed that both the household chief's educational level and the food insecurity of the area account for malnutrition.

**CONCLUSION:**

The height-for-age indicator works better to establish development level. Measures against coverage, relevance and quality of education and access to food can harm the nutritional status of the children.

## INTRODUCTION

According to the Food and Agriculture Organization of the United Nations (FAO), the prevalence of undernutrition increased from 10.6% in 2015 to 10.8% in 2018 worldwide. In South America, it increased from 4.9% in 2015 to 5.5% in 2018; and stunted growth slowly decreases while overweight, obesity and malnutrition quickly increase^1^. In upper-middle-income countries, such as Colombia, stunted growth in under-five was at 6.3%, wasting at 0.6% and overweight at 7.4% in 2018^1^. Colombia faces changes in its economic, demographic and social structures since the 1960s^2^. Consequently, the transition is visible and dynamic regarding variables related to the health^3^, nutritional^4^, and food^5^ status. Despite income increments and poverty reduction in children under five years old (64% in 1999^3^ and 37.2% in 2016^6^), the stunted growth decreased (17.9% in 2000 and 13.2% in 2010^7^) while overweight increased (3.1% in 2000 and 5.2% in 2010^7^).

The development of Colombia has been mediated by armed conflict and forced displacement for the past 60 years^8^^–^^10^. Recently, both advance or retard in the structural and economic development are credited to market economy and trade agreements^11^. According to the report “The Changing Wealth of Nations 2018,” issued by the World Bank^12^, Colombia is the fifth country in the region with the highest wealth per capita and holds one of the most unequal wealth distribution rates in the region: the GINI index was at 0.53 in 2018^3^. The poverty decreased from 37.2% in 2010 to 27.8% in 2015^13^.

The nutritional situation assessed by the *Encuestas Nacionales de Situación Nutricional de Colombia* (ENSIN – Colombia National Survey of the Nutritional Situation) is result of an environment that comprehends the poverty-obesity paradox^14^, the return of communicable diseases and the rise of non-communicable chronic diseases^15^, the increase of income and social tension, the persistence of armed conflict, the enhancement of inequality and differences between the rural and urban development^3^^,^^6^^,^^16^. Thirty two per cent of the Colombian population lives in rural areas where rural social dynamics prevail^9^.

In low- and middle-income countries, despite the gaps between urban and rural, the overweight disparities are reduced. The speed with which overweight occurs is faster in rural areas, while malnutrition in these areas is more relevant than underweight^1^. According to the United Nations (UN), the road map to end the obesity epidemic is multilevel, multidimensional and multisectoral, and it considers the transformation of the food systems throughout education and availability of nutritious and healthy food, both accessible and affordable^17^. Grounded on it, the UN proclamed the Decade of Action on Nutrition in 2016^17^.

This study analyzes the nutritional situation of children under five years old from both urban and rural areas of Colombia.

## METHODS

Analytical study, carried out in Colombia, based on cross-sectional data, collected in the 2015 – 2016 period by ENSIN-2015.

ENSIN were designed to represent 99% of the population, through stratified multistage sampling. The ENSIN-2015 comprehended 44,202 households, representing 4,739 groups from 177 strata. Data from ENSIN-2015 are anonymized, public and can be accessed through a well-founded request to the Colombian Ministry of Health.

The ENSIN-2015 collected anthropometric measurements from 12,908 under-five: 2,484 aged 0–11 months, 2,524 aged 12–23 months, 2,647 aged 24–35 months, 2,567 aged 36–47 months and 2,686 aged 48–59 months. The study population were children under five years old, aged 0–59 months old with admissible anthropometric data. Data from 12,256 children were analyzed.

Trained personnel interviewed the household chief regarding sociodemographic, food security and household economic status. The anthropometric measurements were taken by nutritionists and qualified interviewers, using standard techniques and calibrated equipment. Children under 24 months old had their height taken in supine position^18^^,^^19^. A portable stadiometer was used (Shorr Board Producctions LCC, Olney, MD, USA), and value was rounded to the closest milimeter. Weight was measured by a SECA scale (model no. 874) and value was rounded to 100g divisions. Age was based on date of birth. Age, gender, height and weight data were analyzed in Stata Release 14.1^20^, according to the child growth standards, interpreted by a Z-score classification system, issued by the World Health Organization (WHO)^19^. All children scoring Z<-2 in the indicator weight-for-height fell under the wasting (acute malnutrition) classification. In the same indicator, those scoring Z > 2 were classified as overweight. Children scoring Z<-2 in the height-weight indicator were classified as stunted growth (chronic malnutrition).

The geographic zones were classified according to the population density. The ENSIN-2015 divided the populated centers into four categories, based on their density: a) A Area, 0 – 100,000 inhabitants, b) B Area, 100,000 – 1 million inhabitants, c) C Area, over 1 million inhabitants. These three areas were classified as urban area, and d) “others”, such as the rural area. The “others” category encompassed outskirts close to small cities, rural areas far from small cities and population either spread out or too far from rural areas.

The variables considered were the nutritional situation based on the Z-score, according to the weight-for-height, height-for-age and geographic zone indicators. The covariables: gender, age, household chief's educational level, health insurance, household food security, wealth index, ethnicity and geographic region where individuals live were also considered. The household chief's educational level was based on the formal schooling years attended. The type of health insurance fell under three categories: no health insurance, subsidized coverage, and contributory regime^21^. The food security status was assessed according to the *Escala Latinoamericana y Caribeña de Seguridad Alimentaria* (ELCSA – Latin American and Caribbean Food Security Scale)^22^. The wealth was established according to index designed for international survey on demographics and health^23^. The geographic region is a set of several geodemographic units and, overall, the most relevant aspects of the food culture are represented within them^24^. The occurrence of acute diarrhea and acute respiratory infection two weeks before the survey was self-reported by the household chiefs or their spouses.

Data were analyzed in Stata Release 14.1^20^. The sampling weights of ENSIN-2015 design were incorporated in Stata's complex analysis routines^20^. The analysis was first conducted to identify the demographic structure, through gender and socioeconomic status, within each geographic zone. This established the level of structural and economic development, which determines to great extent the nutritional and health situation of under-fives. The second phase established the raw prevalence, for both wasting and overweight, considering the weight-for-height and height-for-age indicators and each one of the covariables. The third phase of the analysis established Prevalence Ratios (PR), both raw and adjusted, by using the prevalences divided by zone and category of each covariable. For such, the RP and their respective intervals were estimated at 95%CI. The RP were estimated using a binomial regression model having either wasting or overweight (yes/no) as the dependent variable and the geographic zone as the explanatory variable.

Informed consent form was granted during field work. The analyses were compliant with the Declaration of Helsinki^25^. This is a “non-risk” research according to the Resolution no. 8430 from 1993, issued by the Health Ministry of Colombia^26^. Authorization from the Research Ethics Committee of the Universidad Industrial de Santander was not required, since this is a secundary research of population studies, employing anonymized data.

## RESULTS

Prevalence of underweight (weight-for-height < −2) was 1.6% (95%CI 1.3–1.9), showing no difference per zone (p = 0.282) or gender (p = 0.672). The prevalence of overweight (weight-for-height >2) was 5.6% (95%CI 5–6.4), 5.7% (95%CI 5–6.5) in the urban area, 5.5% (95%CI 4.2–7.1) in the rural area, p = 0,787. Overweight had a prevalence of 6.5% (95%CI 5.6–7.6) in men, and 4.7% (4–5.6), p = 0.004 in women. The prevalence of stunted growth (height-for-weight< −2) was 10.8% (95%CI 9.9–11.9), 9% (95%CI 8–10.2) in the urban area and 15.4% (95%CI 13.7–16.3), p < 0.001. Prevalence of stunted growth was 12.2% (95%CI 11–13.5) in men, and 9.5% (95%CI 8.2–10.9), p = 0.004 in women.

Of the children, 71.5% lived in the urban area. The average age was 29.7 months (95%CI 29.3 to 30.2), no difference by gender, p = 0.832. The average age in the urban area was 29.7 months (95%CI 29.1 to 30.3), with no diference by area, which was 29.8 meses (95%CI 29.1 to 30.4), p = 0.911. Distribution by gender and age showed no differences between urban and rural areas; however, economic status, food insecurity (INSA), the educational level of the household chief and coverage of the water supply and sewage networks have showed differences (Table 1).

**Table 1:** Characteristics per geographic area where under-fives live. Colombia, ENSIN-2015.

Variable		Urban area		Rural area		PR (95%CI)^a^	p
		n	Prevalence (95%CI)	n	Prevalence (95%CI)		
Gender							
	Male	4,473	51.5 (49.7–53.3)	1,808	51.4 (49.1–53.6)	1.0 (0.97–1.03)	0.928
	Female	4,378	48.5 (46.7–50.3)	1,752	48.6 (46.4–50.9)	1.0 (0.97–1.03)	0.955
Age group (months)							
	0–11	1,740	20.1 (18.8–21.4)	657	19.3 (17.7–21.1)	1.0 (0.95–1.08)	0.668
	12–23	1,753	19.9 (18.8–21.2)	675	19.8 (18.2–21.5)	1.0 (0.94–1.06)	0.975
	24–35	1,815	19.8 (18.4–21.2)	733	20.9 (18.7–23.3)	1.0 (0.92–1.04)	0.533
	36–48	1,698	19.5 (18.3–20.8)	754	20.7 (18.9–22.6)	1.0 (0.91–1.05)	0.493
	48–59	1,845	20.7 (19.4–22.1)	741	19.3 (17.8–21.0)	1.0 (0.97–1.09)	0.422
Wealth index							
	T_1_- Poorest	1,953	9.6 (8.2–11.3)	2,995	76.9 (72.5–80.8)	0.1 (0.09–0.12)	< 0.001
	T_2_	3,679	37.9 (35.6–40.3)	542	21.7 (18.0–26.0)	1.1 (1.07–1.12)	< 0.001
	T_5_- Wealthiest	3,219	52.5 (50.0–54.9)	23	1.4 (0.9–2.3)	1.0 (1.01–1.02)	< 0.001
Household chief educational level							
	Lower than primary school	1,589	15.4 (14.1–16.8)	1,697	46.6 (42.7–50.6)	0.9 (0.82–0.94)	< 0.001
	Lower than secondary school	2,798	33.0 (31.0–35.1)	1,218	33.7 (31.1–36.4)	1.0 (0.95–1.03)	0.673
	Lower than higher education	3,787	43.8 (41.7–45.9)	583	19.2 (16.4–22.3)	1.1 (1.12–1.17)	< 0.001
	Higher education and further	607	7.8 (6.2–9.8)	27	0.5 (0.3–0.8)	1.0 (0.98–1.07)	0.478
Food insecurity^b^							
	No	3,194	40.4 (38.3–42.6)	969	31.7 (28.6–34.9)	1.1 (1.05–1.13)	< 0.001
	Mild	3,282	37.6 (35.5–39.6)	1,236	35.7 (32.9–38.7)	1.0 (0.99–1.07)	0.234
	Moderate	1,437	13.4 (12.3–14.6)	690	18.3 (16.1–20.7)	0.9 (0.80–0.97)	0.004
	Severe	936	8.6 (7.5–9.9)	660	14.3 (11.9–17.0)	0.8 (0.65–0.90)	< 0.001
Water availability							
	Water supply network	6,171	84.6 (82.3–86.4)	634	22.6 (19.3–26.2)	1.5 (1.42–1.53)	< 0.001
	Well or community supply	353	1.5 (1.04–2.03)	1166	34.0 (29.7–38.7)	0.0 (0.02–0.10)	< 0.001
	Bottled water	1,311	10.0 (8.67–11.6)	161	4.1 (3.05–5.60)	1.1 (1.03–1.12)	0.020
	Others	1,016	3.9 (2.98–5.15)	1,599	39.3 (34.4–44.3)	0.9 (0.84–0.91)	< 0.001
Sewage system							
	Yes	6,924	89.9 (88.0–91.5)	378	16.2 (12.5–20.6)	3.1 (2.96–3.35)	< 0.001
	No	1,927	10.1 (8.5–12.0)	3,182	83.8 (79.4–87.5)	0.0 (0.03–0.05)	< 0.001
Diarrhea^c^							
	Yes	1,057	29.0 (26.6–31.6)	428	38.0 (32.6–43.6)	1.2 (1.08–1.27)	< 0.001
	No	2,314	71.0 (68.4–73.4)	703	62.0 (56.4–67.4)	1.1 (1.06–1.16)	< 0.001
Acute respiratory infection^c^							
	Yes	2,634	79.8 (77.6–81.8)	873	77.6 (72.9–81.6)	1.0 (0.99–1.09)	0.155
	No	737	20.2 (18.2–22.4)	258	22.4 (18.4–27.1)	1.0 (0.88–1.06)	0.441

a PR: prevalente ratio [urban/rural].

b Based on ELCSA.

c Within the previous fifteen days, self-reported.

In the households where the chief had less than high school education, the children living in the rural area presented a smaller rate of poor weight-for-height growth than those living in the urban area, p = 0.013. The same was true for children with no health insurance or under the contributive regime and for those living in places with INSA mild and moderate (Table 2). The children living in households having moderate INSA presented more overweight than those living in the urban area (PR = 3.8; 95%CI 1.7–8.8; p = 0.002). The same was true for those children falling in the lowest tercile of the economic status and those living in the Atlantic region (Table 3).

**Table 2:** Prevalence of wasting in the weight-for-height indicator (Z < −2) in Colombian children aged between zero and four years per geographic area. Colombia, ENSIN-2015.

Variable		Urban area (n = 8,735)		Rural area (n = 3,521)		aPR^a^ (95%CI)	p
		Prevalence (95%CI)	p	Prevalence (95%CI)	p		
Total		1.4 (1.13–1.83)		1.8 (1.29–2.57)	0.282	0.7 (0.44–1.10)	0.120
	Gender		0.753		0.766		
	Male	1.5 (1.12–2.00)		1.9 (1.13–3.25)		0.8 (0.40–165)	0.563
	Female	1.4 (0.94–2.04)		1.7 (1.07–2.76)		0.6 (0.31–1.10)	0.093
Age group (months)			0.001		0.004		
	0–11	2.4 (1.64–3.47)		2.9 (1.31–6.50)		0.6 (0.25–1.37)	0.211
	12–23	1.7 (1.12–2.61)		2.7 (1.41–4.98)		0.5 (0.25–1.13)	0.099
	24–35	1.5 (0.75–3.19)		1.5 (0.60–3.70)		0.9 (0.33–2.34)	0.790
	36–48	0.5 (0.02–1.02)		1.9 (1.00–3.72)		4.7 (0.65–33.65)	0.124
	48–59	1.1 (0.67–1.86)		0.0 (0.00–0.34)		0.0 (0.00–0.32)	0.004
Stunted growth (height-for-age)			0.272		0.798		
	No	1.5 (1.16–1.91)		1.8 (1.21–2.66)		0.7 (0.41–1.36)	0.139
	Yes (Z < −2)	1.0 (0.50–1.98)		2.0 (1.06–3.65)		0.7 (0.28–1.91)	0.511
Household chief's educational level			0.984		0.058		
	Lower than primary school	1.5 (0.90–2.54)		2.7 (1.76–4.06)		1.0 (0.44–2.14)	0.944
	Lower than secondary school	1.2 (0.80–1.75)		0.9 (0.37–2.29)		0.3 (0.12–0.77)	0.013
	Lower than higher education	1.8 (1.24–2.61)		1.3 (0.57–3.12)		0.6 (0.22–1.62)	0.303
	Higher education or further	0.6 (0.25–1.24)		NO		NA	NA
Health insurance			0.009		< 0.001		
	Uninsured	1.1 (0.64–1.70)		0.2 (0.00–0.73)		0.1 (0.04–0.31)	< 0.001
	Subsidized	1.7 (1.26–2.19)		2.2 (1.51–3.13)		0.8 (0.50–1.40)	0.490
	Contributive/others	4.1 (2.12–5.46)		1.5 (0.47–4.57)		0.2 (0.05–0.84)	0.029
Food insecurity^b^			0.114		0.132		
	No	1.2 (0.68–1.99)		1.7 (0.84–3.30)		1.1 (0.45–2.57)	0.872
	Mild	1.5 (1.09–2.10)		1.2 (0.69–2.12)		0.4 (0.19–0.97)	0.043
	Moderate	1.5 (0.84–2.67)		1.5 (0.72–3.08)		0.4 (0.14–0.92)	0.034
	Severe	2.3 (1.34–4.02)		4.1 (1.99–8.34)		0.8 (0.38–1.58)	0.476
Wealth index			0.010		0.077		
	T_1_- poorest	2.8 (1.76–4.60)		2.2 (1.54–3.19)		0.7 (0.40–1.26)	0.239
	T_2_	1.6 (1.16–2.19)		0.6 (0.12–2.58)		0.4 (0.07–1.70)	0.190
	T_5_- wealthiest	1.1 (0.67–1.70)		NO		NA	NA
Ethnicity			0.119		0.174		
	Mestizo	1.3 (1.01–1.76)		1.6 (1.13–2.31)		0.6 (0.36–1.05)	0.072
	Black/Afro-descendant	2.5 (1.47–4.33)		1.1 (0.41–2.77)		0.3 (0.07–1.58)	0.152
	Indigenous	1.0 (0.32–3.37)		3.6 (1.41–8.92)		1.5 (0.44–5.33)	0.473
Region			0.116		0.068		
	Central	1.1 (0.67–1.76)		2.4 (1.45–4.01)		0.7 (0.28–1.55)	0.318
	Atlantic	2.3 (1.69–3.03)		2.2 (1.05–4.59)		0.5 (0.25–1.05)	0.067
	Oriental	1.8 (0.79–3.85)		0.9 (0.50–1.65)		1.5 (0.63–3.34)	0.350
	Pacific	1.2 (0.65–2.26)		1.8 (0.95–3.40)		1.0 (0.23–4.26)	0.994
	Bogotá	0.9 (0.41–1.99)		NO		NA	NA
	Amazon/Orinoquia	0.6 (0.31–1.15)		NO		NA	NA

NO: no occurences; NA: not available.

a aPR: adjusted prevalence ratio [rural/urban] found in a binomial model, having the wasting prevalence in the weight-for-height indicator, Z < −2, as the independent variable and area as the explanatory variable. Adjustment was applied by the following covariables: gender, age, stunted growth in height-for-age, Z < −2, household chief's educational level, health insurance status, household food insecurity, wealth index, ethnicity and region

b Based on ELCSA.

**Table 3:** Prevalence of overweight in the weight-for-height indicator (Z > 2) in Colombian children aged between zero and four years per geographic area. Colombia, ENSIN-2015.

Variable		Urban area (n = 8,735)		Rural area (n = 3,521)		aPR^a^ (95%CI)	p
		Prevalence (95%CI)	p	Prevalence (95%CI)	p		
Total		5.7 (4.98–6.52)		5.5 (4.20–7.12)	0.672	1.3 (0.83–2.01)	0.244
Gender			0.013		0.093		
	Male	6.6 (5.52–7.93)		6.3 (4.64–8.54)		1.3 (0.82–2.11)	0.251
	Female	4.7 (3.90–5.73)		4.6 (3.24–6.48)		1.3 (0.74–2.27)	0.360
Age group (months)			0.182		0.001		
	0–11	6.2 (4.63–8.32)		6.7 (4.56–9.80)		1.2 (0.73–1.99)	0.460
	12–23	6.2 (4.76–8.01)		7.9 (4.72–12.9)		2.0 (0.91–4.51)	0.081
	24–35	6.5 (4.75–8.85)		5.3 (3.40–8.14)		0.8 (0.41–1.74)	0.639
	36–48	4.1 (3.07–5.49)		5.4 (3.02–9.47)		2.4 (0.80–7.00)	0.119
	48–59	5.5 (4.09–7.32)		2.0 (1.08–3.81)		0.5 (0.21–1.34)	0.178
Stunted growth (hight-for-age)			0.132		0.239		
	No	5.5 (4.77–6.35)		5.2 (4.10–6.53)		1.2 (0.85–1.82)	0.247
	Yes (Z < −2)	7.7 (5.06–11.5)		7.1 (3.86–12.7)		1.8 (0.57–5.78)	0.302
Household chief's educational level			0.351		0.995		
	Lower than primary school	5.9 (4.24–8.19)		5.2 (3.13–8.65)		1.4 (0.31–6.32)	0.656
	Lower than secondary school	5.1 (4.03–6.42)		5.8 (4.23–7.90)		1.4 (0.92–2.25)	0.107
	Lower than higher education	5.8 (4.81–7.00)		5.3 (3.49–7.86)		1.1 (0.68–1.89)	0.616
	Higher education or further	7.9 (4.56–13.4)		NO		NA	NA
Health insurance			0.003		0.754		
	Uninsured	7.0 (5.81–8.43)		6.3 (3.46–11.1)		0.8 (0.45–1.52)	0.562
	Subsidized	4.3 (3.52–5.17)		5.3 (3.81–7.23)		1.7 (0.85–3.43)	0.133
	Contributive/others	5.5 (3.00–9.78)		6.2 (3.58–10.4)		2.0 (0.46–8.73)	0.342
Food insecurity^b^			0.024		0.641		
	No	7.2 (5.86–8.88)		5.0 (3.69–6.81)		0.7 (0.48–1.18)	0.210
	Mild	4.3 (3.46–5.38)		6.2 (4.50–8.56)		1.5 (0.96–2.36)	0.077
	Moderate	6.1 (4.39–8.48)		6.6 (2.54–15.9)		3.8 (1.67–8.80)	0.002
	Severe	4.0 (2.43–6.43)		3.2 (2.01–5.23)		1.2 (0.52–2.82)	0.649
Wealth index			< 0.001		0.677		
	T_1_- poorest	2.6 (1.69–3.85)		5.2 (4.23–6.33)		2.0 (1.30–3.19)	0.002
	T_2_	5.1 (4.17–6.25)		6.8 (3.01–14.6)		1.2 (0.50–3.04)	0.649
	T_5_- wealthiest	6.7 (5.57–8.07)		1.5 (0.20–9.97		0.2 (0.03–1.38)	0.102
Ethnicity			0.097		0.359		
	Mestizo	5.8 (5.02–6.71)		5.7 (4.12–7.78)		1.3 (0.75–2.15)	0.367
	Black/Afro-descendant	4.9 (3.20–7.48)		5.1 (3.19–8.08)		1.9 (0.75–4.72)	0.162
	Indigenous	2.4 (1.42–4.19)		4.2 (2.41–7.25)		2.3 (0.98–5.40)	0.056
Region			0.704		0.121		
	Central	6.5 (5.04–8.39)		7.5 (4.00–13.7)		1.8 (0.54–6.19)	0.306
	Atlantic	5.0 (4.14–6.05)		4.4 (3.09–6.12)		2.4 (1.43–3.95)	0.003
	Oriental	5.8 (3.91–8.64)		6.2 (3.91–9.71)		0.7 (0.28–1.53)	0.305
	Pacific	5.3 (3.63–7.81)		4.8 (3.36–6.69)		1.2 (0.52–2.60)	0.680
	Bogotá	5.7 (3.95–8.27)		NO		NA	NA
	Amazon/Orinoquia	5.9 (4.59–7.48)		0.1 (0.00–1.97)		7.6 (1.29–44.86)	0.011

NO: no occurences; NA: not available.

a aPR: adjusted prevalence ratio [rural/urban] found in a binomial model, having the wasting prevalence in the weight-for-height indicator, Z < −2, as the independent variable and area as the explanatory variable. Adjustment was applied by the following covariables: gender, age, stunted growth in height-for-age, Z < −2, household chief's educational level, health insurance, household food insecurity, wealth index, ethnicity and region

b Based on ELCSA.

The stunted growth was greater in the rural area (PR = 1.24; 95%CI 1–1.53; p = 0.050); and the same was found by comparing the prevalence of poor weight-for-height growth in the rural area against some categories of covariables; while those under one year old, overweight children whose parents had a low educational level, those living in households with moderate INSA and in the central region presented greater stunted growth rates if living in the rural area (Table 4).

**Table 4:** Prevalence of stunted growth in the height-for-age indicator (Z > 2) in Colombian children aged between zero and four years per geographic area. Colombia, ENSIN-2015.

Variable		Urban area (n = 8,735)		Rural area (n = 3,521)		aPR^a^ (95%CI)	p
		Prevalence (95%CI)	p	Prevalence (95%CI)	p		
Total		9.0 (7.96–10.2)		15.4 (13.7–17.3)	0.004	1.2 (1.00–1.53)	0.050
Gender			0.041		0.033		
	Male	10.0 (8.67–11.6)		17.4 (15.1–20.0)		1.2 (0.97–1.59)	0.087
	Female	7.9 (6.52–9.61)		13.3 (10.8–16.3)		1.2 (0.85–1.84)	0.246
Age group (months)			0.102		0.010		
	0–11	5.4 (3.14–9.17)		8.1 (5.32–12.1)		2.2 (1.20–3.89)	0.011
	12–23	9.4 (7.31–12.1)		16.2 (12.3–21.2)		1.4 (0.81–2.52)	0.220
	24–35	12.3 (10.0–14.9)		21.2 (17.3–25.7)		0.9 (0.68–1.26)	0.615
	36–48	9.2 (7.43–11.3)		16.7 (13.2–21.0)		0.3 (0.83–2.15)	0.227
	48–59	8.8 (7.04–10.9)		14.2 (11.1–18.1)		1.2 (0.80–1.72)	0.401
Nutrition situation (weight-for-height)			< 0.001		< 0.001		
	Acute malnutrition (Z < −2)	6.2 (3.14–12.0)		16.7 (8.76–2.94)		1.6 (0.40–6.20)	0.491
	Normal	8.9 (7.80–10.1)		15.1 (13.3–17.1)		1.2 (0.97–1.51)	0.093
	Oerweight (Z > 2)	12.2 (8.04–18.0)		20.0 (12.5–30.4)		1.7 (1.30–2.33)	< 0.001
Household chief's educational level			< 0.001		0.020		
	Lower than primary school	10.5 (8.14–13.4)		18.3 (15.7–21.2)		1.7 (1.21–2.50)	0.003
	Lower than secondary school	11.4 (9.33–13.9)		13.0 (10.4–16.2)		0.8 (0.58–1.20)	0.326
	Lower than higher education	7.3 (6.15–8.72)		12.4 (8.02–18.6)		1.5 (1.03–2.10)	0.034
	Higher education or further	4.8 (2.76–8.09)		11.8 (2.22–44.0)		2.3 (0.87–6.00)	0.089
Health insurance status			0.011		0.639		
	Uninsured	7.3 (5.78–9.16)		13.3 (9.04–19.2)		1.8 (1.07–2.94)	0.027
	Subsidized	10.5 (9.17–11.9)		16.0 (14.1–18.1)		1.1 (0.90–1.42)	0.301
	Contributive/others	9.0 (5.97–13.5)		12.9 (8.48–19.2)		NA	NA
Food insecurity^b^			0.081		< 0.001		
	No	8.6 (6.76–10.8)		10.2 (7.96–13.0)		0.8 (0.53–1.19)	0.252
	Mild	8.1 (6.72–9.84)		13.1 (10.3–16.5)		1.1 (0.79–1.65)	0.470
	Moderate	11.0 (8.92–13.5)		18.8 (14.7–23.6)		1.7 (1.09–2.59)	0.019
	Severe	11.8 (8.72–15.8)		28.4 (24.3–33.0)		1.5 (1.01–2.26)	0.044
Wealth index			0.001		0.089		
	T_1_- poorest	13.5 (10.8–16.8)		16.3 (14.5–18.3)		1.2 (0.91–1.52)	0.203
	T_2_	10.0 (8.61–11.6)		12.5 (7.99–19.0)		1.3 (0.82–2.03)	0.264
	T_5_- wealthiest	7.5 (5.96–9.32)		11.1 (1.58–49.1)		NA	NA
Ethnicity			0.720		< 0.001		
	Mestizo	9.0 (7.88–10.3)		12.8 (11.0–14.9)		1.0 (0.79–1.35)	0.796
	Black/Afro-descendant	5.7 (3.78–8.60)		10.7 (7.97–14.3)		1.4 (0.68–3.00)	0.319
	Indigenous	17.8 (11.9–25.8)		34.0 (29.1–39.3)		1.3 (0.75–2.34)	0.312
Region			0.008		0.337		
	Central	7.1 (5.53–9.15)		15.5 (12.1–19.5)		2.0 (1.02–3.76)	0.043
	Atlantic	8.7 (7.38–10.3)		19.4 (16.7–22.4)		1.0 (0.73–1.31)	0.863
	Oriental	8.3 (5.96–11.5)		11.6 (8.93–14.8)		0.9 (0.55–1.65)	0.840
	Pacific	7.5 (5.55–10.1)		13.9 (9.77–19.4)		1.4 (0.71–2.70)	0.303
	Bogotá	13.1 (9.43–17.9)		NO		NA	NA
	Amazon/Orinoquia	10.4 (8.93–12.1)		16.2 (6.23–36.1)		NA	NA

NO: no occurences; NA: not available.

a aPR: adjusted prevalence ratio [rural/urban] found in a binomial model, having the wasting prevalence in the weight-for-height indicator, Z < −2, as the independent variable and area as the explanatory variable. Adjustment was applied by the following covariables: gender, age, stunted growth in height-for-age, Z < −2, household chief's educational level, health insurance, household food insecurity, wealth index, ethnicity and region

b Based on ELCSA.

## DISCUSSION

Based on ENSIN-2015, the prevalence of wasting and overweight was estimated in under-fives with the weight-for-height indicator and stunted growth with the height-for-age indicator. The prevalence of stunted growth increased from 13.2% in 2010^7^ to 10.8% in 2015. However, both wasting and overweight increased, with acute malnutrition going from 0.9% in 2010^7^ to 1.6% in 2015 and overweight from 5.2% in 2010^7^ to 5.6% in 2015. In addition, geographic zone had not presented statistical significance to the prevalences of wasting and overweight in the weight-for-height indicador, but rather had presented it to the height-for-age indicator.

The weight-for-height indicator in Colombia is smaller than the average reported by FAO for 2018 in Asian countries, which is 22.7%, or in African countries, which is 30%, but higher than average in high-income countries, which is 3% higher, and in Latin American, which is of 9%. The overweight (weight-for-heigh) rate in Colombia is higher than the Asian countries average of 2.2%, or the 4.9% in African coutries, and smaller than the Latin America average of 7.5%, and than high-income countries, such as the United States, which is 7.2%.

According to the World Bank and the United Nations Children's Fund (UNICEF), the prevalence of stunted growth is decreasing in all upper-middle-income countries of the region, such as Colombia^27^. The last figures available on under-fives and supplied by national surveys are slightly close to figures in this study, disclosing rates of 8.2% in Argentina (2005), 5.6% in Costa Rita (2009), 7% in Cuba (2000), 7.1% in Dominican Republic (2013), 23.9% in Ecuador (2014), 6.2% in Jamaica (2014), 6.2% in Mexico (2016), 5.6% in Paraguay (2016), 13.1% in Peru (2016), 13.4 % in Venezuela (2009)^27^.

Additionally, the overweight in the same indicator is slightly gerater than reported by this study than rates reported in high-income countries: 9.9% in Argentina (2005), 8.1% in Costa Rita (2009) 8,1%, 7.6% in Dominican Republic (2013), 8% in Ecuador (2014), 8.5% in Jamaica, 5.2% in México (2016), 6.2% in Panama (1997), 12.4% in Paraguay (2016), 7.2% in Peru (2016), and 6.4% in Venezuela (2009) (46). The prevalence of wasting in the weight-for-heigh indicator is under the threoretical 2.5% of these countries. In the United States, a high-income country, the stunted growth is rated at 2.1% and overweight at 6%^27^.

The classification of urban and rural areas in the ENSIN-2015 is based on the concentration of population unaware of the territorial concept and of the seven dimensions constitutive of it^9^^,^^28^. Yet, the rural concept, as tradicionally known, encompasses the relationships of the individuals living there, which are predominantly towards farming^2^^,^^28^. Poverty and other variables – here the nutritional situation – reaching similar figures in rural and urban areas are theoretically differents, given they resulted from structurally dissimilar processes in the geographic zone. For example, a) goods and services are more expensive in urban than rural area, b) urban survival requires goods and services not needed in the rural environment, c) money is more important in the urban than rural area, d) urban households are more vunerable than those of the rural areas; e) the position of the household chief in urban area is determined by the labor market, whereas the same does not occurr in rural area, f) development impacts urban areas, whereas has little or no impacto in rural areas and g) access to food in urban areas relies almost exclusively on money, whereas the same does not occurr in the rural areas. Besides, we established that the educational level of the household chief and of household's INSA, in addition to basic infrastructure variables, such as water supply and sewage networks availability, are different in each geographic zone (Table 1).

Regarding to the wasting in the weight-for-heigh indicator, the educational level of the household chief – less than high school –, type of health insurance – no health insurance or contributive regime –, and INSA – mild or moderate, were associated as expected. The PR in these variables show that acute malnutrition is smaller in the rural area. When prevalences are adjusted, despite reduced individual capacities, such as education^29^ or institutionality, such as health insurance, a smaller wasting rate in the weight-for-heigh indicator is associated with mild and moderate INSA in rural areas, rather than the urban ones (Table 2). This finding diverges from what we already know about the Colombian set and invites us to rethink the inequalities by geographic zone. The current dynamics in study variables become potencial confounders of the wasting prevalence.

Those classified as poorer by the economic index, the households with mild INSA and the Atlântico region, of which poor departments such as La Guajira are part, presented overweight rates higher in rural than in urban areas (Table 3). The overweight associated with greater poverty was observed when the nutrition transition was declared in Latin America^14^, with the ENSIN-2010^4^ data in Colombia and in Colombian children^30^.

The poor growth in the height-for-weight indicator corroborates that minor determinants of social development (the educational level of the household chief), which lead to reduced food access (INSA), combined to an enviroment of institutional inequalities (reduced health coverage), are shown at an early age (in children under one year old) in the form of stunted growth. Biologically, the increased obesity vicious cycle in rural children, along with stunted growth, illustrate what was already described in the 1960s^31^.

The Colombian rural area presents chronics inequalities, which strongly limitate the human, social, structural and economic development; their effects being visibles on both individual and collective levels. This chronic state of urban underdevelopment is also evidenced by the nutritional situation, and even though poverty was reduced, the same is not true for inequality. The develpment and underdevelopment ways change and yet, the nutritional situation remains sensitive to these. The greater contribution of this study is not whether there are inequalities between rural and urban areas, as for over 70 years these are indeniable in Colombia. The evidences are shown in all countries in the region, including Brazil^32^ and other low-income countries^33^. Its greatest contribution is to establish a set of variables expressing the edges of development in Colombia, factors that currently affect the nutrition situation. To increase individual capacities – through education as a mid and long-term strategy – and to guarantee food access – using short, mid and long-term measures – are the key variables to positively impact the nutrition situation of children under five years and general population. This corroborates both the intent of the Colombian state^2^^,^^3^^,^^28^ and the UN^17^.

The analyzed data were extracted from a national survey and through multivariate analysis, and the potential confounder effect of variables traditionally associated with the nutrition situation of under-five was cleared. The methods used to determine both the nutrition status and geographic zones classifications are universally accepted and remain sensitive to inequality under the current conditions and level of development reached in Colombia. The main limitation found is the cross-sectional data source. However, the coherence with the knowledge accumulated over the past 100 years assures external validity to the study.

The prevalence of nutrition situation in the indicators follows the regional and global trends. Geographic zones cause no differences in the prevalences of wasting and overweight in the weight-for-height indicator, only in the height-for-age indicator. Height-for-age is the best indicator to establish the level of geographic development and the gaps between rural and urban areas. Some expressions of the socioeconomic development were related to the nutrition situtation, being the household chief educational level and household food insecurity the most relevant. The nutrition transition in Colombia is dynamic. These findings will enable the design and adjustment of related public policies to achieve cost-effectiveness.
