# Supplementary material for: Nutrition gap in children, urban-rural: the key education and food. Colombia, 2015
Source: Rev Saude Publica. 2020 Nov 4;54:111. doi: 10.11606/s1518-8787.2020054001925 (PMC7647466; doi:10.11606/s1518-8787.2020054001925)
Supplement: Translation

# Translation: Brecha nutricional en niños, urbano-rural: educación y alimentos, la clave. Colombia, 2015

**Authors:** Jhael N. Bermúdez, Daniel Ayala, Oscar F. Herrán

**Affiliations:** I BogotáD.C Colombia Instituto Colombiano de Bienestar Familiar. Subdirección de Monitoreo y Evaluación. Bogotá D.C, Colombia; II Chile Pontificia Universidad Católica de Chile; III Bucaramanga Colombia Universidad Industrial de Santander. Escuela de Nutrición y Dietética. Bucaramanga, Colombia

**Keywords:** Preescolar, Encuestas Nutricionales, Seguridad Alimentaria y Nutricional, Disparidades en el Estado de Salud, Medio Rural, Área Urbana

## Abstract

**OBJETIVO:**

Analizar el estado de nutrición en menores de cinco años de áreas urbanas y rurales en Colombia.

**MÉTODOS:**

Estudio analítico, con base en datos de corte transversal, recolectados por la ENSIN-2015. La muestra fue de 12.256 niños colombianos entre cero y cuatro años. Se calcularon razones de prevalencia (RP) y sus respectivos intervalos al 95% de confianza (IC95%). Las RP se obtuvieron de modelos de regresión binomial con el déficit o el exceso, como la variable dependiente y la zona geográfica como la principal explicación. Variables del contexto se utilizaron para ajustar las RP estimadas y limpiar el efecto confusor de éstas.

**RESULTADOS:**

La prevalencia de desnutrición aguda (peso/talla) fue de 1,6%, la de exceso de 5,6%. No existieron diferencias por zona geográfica, en el indicador (peso/talla). El retraso talla/edad – desnutrición crónica – fue mayor en la zona rural (RP = 1,2; IC95% 1,00–1,53; p = 0,050). Las prevalencias ajustadas por variables que dan cuenta del desarrollo estructural, social y económico, mostraron que la escolaridad del jefe y la inseguridad alimentaria del hogar explican la desnutrición.

**CONCLUSIONES:**

El indicador talla/edad es el mejor para establecer el nivel de desarrollo. Medidas contra la cobertura, pertinencia, calidad en la educación y el acceso a los alimentos impactarán negativamente el estado de nutrición en los niños.

## INTRODUCCIÓN

Según la Organización de la Naciones Unidas para la Agricultura (FAO), la prevalencia de subalimentación aumentó en el mundo de 10,6% en 2015 a 10,8% en 2018. En América del Sur, pasó de 4,9% en 2015 y a 5,5% en 2018; el retraso del crecimiento en los niños disminuye lentamente y el exceso de peso, la obesidad y la malnutrición aumentan rápidamente^1^. En países de ingresos medianos altos, como Colombia, el retraso del crecimiento en los menores de cinco años era del 6,3%, la emaciación del 0,6% y el sobrepeso de 7,4% en 2018^1^. Colombia experimenta cambios en su estructura económica, demográfica y social desde los 60´s^2^. Como consecuencia, el fenómeno de transición es visible y dinámico en variables que dan cuenta del estado de salud^3^, nutrición^4^ y alimentación^5^. A pesar del aumento en el ingreso y la disminución de la pobreza en los menores de cinco años (64,0% en 1999^3^ y 37,2% en 2010^6^), disminuyó el retraso del crecimiento (17,9% en 2000 y 13,2% en 2010^7^) pero aumentó el exceso de peso (3,1% en 2000 y 5,2% en 2010^7^).

Las expresiones del desarrollo en Colombia han sido mediadas por el conflicto armado y el desplazamiento forzado durante los últimos 60 años^8^^–^^10^. Más recientemente, a la economía de mercado y al comercio regulado por tratados, se le atribuye el avance o retraso en el desarrollo estructural y económico^11^. Según el informe “La riqueza cambiante de las naciones 2018”, elaborado por el Banco Mundial^12^, Colombia es el quinto país en la región con más riqueza *per cápita*, y uno de los más desiguales en la distribución de la riqueza en la región: el coeficiente de concentración de la riqueza, establecido por el estadístico Coreano Gini (GINI) era de 0,53 en 2018^3^. La pobreza disminuyó de 37,2% en 2010 a 27,8% en 2015^13^.

El estado de nutrición establecido a través de las Encuestas Nacionales de la Situación Nutricional en Colombia (ENSIN) es el resultante de un entorno donde ocurre obesidad en la pobreza^14^, existe re emergencia de enfermedades transmisibles y aumentan las enfermedades crónicas no transmisibles^15^, aumenta el ingreso y la tensión social, persiste el conflicto armado, la inequidad y las diferencias en el desarrollo rural y urbano se acentúan^3^^,^^6^^,^^16^. El 32,0% de la población colombiana es rural y priman las relaciones de sociedades rurales^9^.

A pesar de las brechas entre urbano y rural, en los países de ingresos medianos y bajos, las diferencias en el exceso de peso se reducen. La velocidad con que se pasa al exceso de peso es mayor en las áreas rurales y la malnutrición en éstas es más importante que el déficit de peso^1^. Según las Naciones Unidas (ONU), la hoja de ruta para revertir la epidemia de obesidad es multinivel, multidimensional y multisectorial y considera la transformación de los sistemas alimentarios a través de la educación y con disponibilidad de alimentos nutritivos y saludables que sean fáciles de conseguir y asequibles^17^. Con base en lo anterior, la ONU proclamó el Decenio de Acción sobre la Nutrición en 2016^17^.

El objetivo del estudio fue analizar el estado de nutrición en menores de cinco años de áreas urbanas y rurales en Colombia.

## MÉTODOS

Estudio analítico, realizado en Colombia con base en datos de corte transversal, recolectados en el período 2015–2016 por la ENSIN-2015.

Las ENSIN son diseñadas para representar al 99,0% de la población, a través de muestreo polietápico estratificado. La ENSIN-2015 incluyó 44.202 hogares, representando 4.739 clúster de 177 estratos. Los datos de la ENSIN-2015 están anonimizados, son públicos y se pueden obtener mediante solicitud razonada al Ministerio de Salud de Colombia.

La ENSIN-2015 tomó datos antropométricos a 12.908 menores de cinco años: 2.484 entre cero y 11 meses, 2.524 entre 12 y 23 meses, 2.647 entre 24 y 35 meses, 2.567 entre 36 y 47 meses y 2.686 entre 48 y 59 meses de edad. La población objeto de estudio fue la de menores entre cero y 59 meses con datos plausibles de antropometría. Datos de 12.256 niños fueron analizados.

Personal entrenado administró al jefe del hogar cuestionarios para obtener información sociodemográfica, sobre seguridad alimentaria y el nivel de riqueza del hogar. Las medidas antropométricas fueron tomadas por nutricionistas y encuestadores entrenados, utilizando técnicas estandarizadas y equipos calibrados. Los niños menores de 24 meses fueron tallados en posición decúbito supino^18^^,^^19^. La talla fue establecida con tallímetro portátil (*Shorr Board Producctions LCC, Olney*, *MD, USA*), aproximando al milímetro más cercano. El peso fue establecido con básculas SECA (modelo 874), aproximando a 100 g. La edad fue establecida con base en la fecha de nacimiento. Los datos de edad, sexo, talla y peso fueron procesados con los macros del software Stata versión 14,1^20^ y con base en el patrón internacional de referencia del crecimiento y desarrollo de la OMS calculadas puntuaciones Z estandarizadas^19^. El déficit en el indicador peso/talla (P/T) (desnutrición aguda) se declaró para todos los niños con Z < −2. El exceso de peso en el mismo indicador se declaró para todos los niños con Z > 2. El retraso en la talla (desnutrición crónica) se declaró para todos los niños con Z < −2 en talla/edad (T/E)..

La zona geográfica se declaró con base en la concentración de la población. La ENSIN-2015 clasificó en cuatro categorías a los centros poblados con base en la concentración de la población: a) cabecera A, de cero a 100 mil habitantes, b) cabecera B, de 100 mil a un millón de habitantes, c) cabecera C, a las poblaciones con más de un millón de habitantes. Las tres cabeceras fueron declaradas la zona urbana, y d) la categoría “resto”, como la zona rural. La categoría “resto” incluyó centros poblados suburbanos cercanos a ciudades pequeñas, cabeceras de áreas rurales distantes de ciudades pequeñas y población dispersa o muy distante de cabeceras rurales.

Las variables de interés fueron la clasificación del estado nutricional con base en puntuaciones Z estandarizadas, según los indicadores P/T, T/E y la zona geográfica. También se consideraron otras covariables: el sexo, la edad, la escolaridad del jefe del hogar, el tipo de aseguramiento al régimen de salud, el estado de seguridad alimentaria en el hogar, el índice de riqueza, la etnia y la región geográfica donde habitan los sujetos. La escolaridad del jefe del hogar se clasificó con base en los años formales de estudio aprobados. El tipo de aseguramiento al sistema de salud y protección social en salud se clasificó en tres: sin aseguramiento, subsidiado y régimen contributivo^21^. El estado de seguridad alimentaria del hogar fue establecido a través de la Escala Latinoamericana y Caribeña de Seguridad Alimentaria (ELCSA)^22^. La riqueza fue establecida con el índice diseñado para la encuesta internacional de demografía y salud^23^. La región geográfica es una agrupación de varias unidades geodemográficas y en general, éstas comparten en su interior los aspectos más relevantes de la cultura alimentaria^24^. La presencia de enfermedad diarreica aguda (EDA) e infección respiratoria aguda (IRA) en los últimos 15 días se obtuvo por auto reporte del jefe del hogar o su cónyuge.

Los análisis fueron conducidos utilizando del software Stata, versión 14,1^20^. El peso muestral del diseño de la ENSIN-2015 fue incorporado en las rutinas de análisis complejos de Stata^20^. El análisis se condujo en primera instancia para describir en la zona geográfica la estructura demográfica, por sexo, las condiciones sociales y económicas. Eso permitió establecer el grado de desarrollo estructural y económico, que a su vez, determina en buena medida el estado de salud y nutrición de los menores de cinco años. La segunda fase del análisis estuvo dirigida a establecer las prevalencias crudas, tanto de déficit como de exceso, en los indicadores P/T y T/E y para cada una de las categorías en las covariables. La tercera fase del análisis se orientó para establecer las razones de prevalencia (RP) crudas y ajustadas entre prevalencias por zona y categoría de cada covariable. Para esto, se calcularon RP y sus respectivos intervalos al 95% de confianza (IC95%). Las RP se obtuvieron de modelos de regresión binomial con el déficit o el exceso (si/no), como la variable dependiente y la zona geográfica como la principal explicación. Las RP ajustadas y sus respectivos IC95% incorporaron el diseño complejo de la muestra.

El consentimiento y asentimiento para participar fue obtenido durante el operativo de campo. Los análisis se realizaron bajo los principios de la Declaración de Helsinki^25^. Esta investigación se clasifica como “sin riesgo” según la Resolución 8.430 de 1993, MinSalud, Colombia^26^. Por ser análisis secundarios de estudios poblacionales, con datos anonimizados, no se requiere autorización del Comité de Ética en Investigación en Salud de la Universidad Industrial de Santander.

## RESULTADOS

La prevalencia de bajo peso para la talla (P/T < −2) fue de 1,6% (IC95% 1,3–1,9), sin diferencia por zona (p = 0,282) o sexo (p = 0,672). La prevalencia de exceso de peso para la talla (P/T>2), fue de 5,6% (IC95% 5,0–6,4), en la zona urbana de 5,7% (IC95% 5,0–6,5), en la zona rural de 5,5% (IC95% 4,2–7,1), p = 0,787. El exceso de peso en los hombres fue de 6,5% (IC95% 5,6–7,6), en las mujeres de 4,7% (4,0–5,6), p = 0,004. La prevalencia de retraso de talla para la edad (T/E < −2), fue de 10,8% (IC95% 9,9–11,9), en la zona urbana de 9,0% (IC95% 8,0–10,2), en la zona rural de 15,4% (IC95% 13,7–16,3), p < 0,001. El retraso en talla en los hombres fue de 12,2% (IC95%11,0–13,5), en las mujeres de 9,5% (IC95% 8,2–10,9), p = 0,004.

El 71,5% de los niños habitaban en la zona urbana. La media de edad de los sujetos fue de 29,7 meses (IC95% 29,3–30,2), sin diferencia por sexo, p = 0,832. La distribución por sexo y de la estructura de edad no fueron diferentes entre zona urbana y rural, pero sí hubo diferencias en el nivel de riqueza, en la inseguridad alimentaria (INSA), en la escolaridad del jefe del hogar y en la cobertura de servicios de acueducto y alcantarillado (Tabla 1).

**Tabla 1:** Características por área geográfica donde habitan menores de cinco años. Colombia, ENSIN–2015.

Variable		Urbano		Rural		RP (IC95%)^a^	p
		n	Prevalencia (IC95%)	n	Prevalencia (IC95%)		
Sexo							
	Hombre	4.473	51,5 (49,7–53,3)	1.808	51,4 (49,1–53,6)	1,0 (0,97–1,03)	0,928
	Mujer	4.378	48,5 (46,7–50,3)	1.752	48,6 (46,4–50,9)	1,0 (0,97–1,03)	0,955
Grupo de edad (meses)							
	0–11	1.740	20,1 (18,8–21,4)	657	19,3 (17,7–21,1)	1,0 (0,95–1,08)	0,668
	12–23	1.753	19,9 (18,8–21,2)	675	19,8 (18,2–21,5)	1,0 (0,94–1,06)	0,975
	24–35	1.815	19,8 (18,4–21,2)	733	20,9 (18,7–23,3)	1,0 (0,92–1,04)	0,533
	36–48	1.698	19,5 (18,3–20,8)	754	20,7 (18,9–22,6)	1,0 (0,91–1,05)	0,493
	48–59	1.845	20,7 (19,4–22,1)	741	19,3 (17,8–21,0)	1,0 (0,97–1,09)	0,422
Índice de riqueza							
	T_1_- los más pobres	1.953	9,6 (8,2–11,3)	2.995	76,9 (72,5–80,8)	0,1 (0,09–0,12)	< 0,001
	T_2_	3.679	37,9 (35,6–40,3)	542	21,7 (18,0–26,0)	1,1 (1,07–1,12)	< 0,001
	T_5_- los más ricos	3.219	52,5 (50,0–54,9)	23	1,4 (0,9–2,3)	1,0 (1,01–1,02)	< 0,001
Escolaridad del jefe							
	Menos de primaria	1.589	15,4 (14,1–16,8)	1.697	46,6 (42,7–50,6)	0,9 (0,82–0,94)	< 0,001
	Menos de secundaria	2.798	33,0 (31,0–35,1)	1.218	33,7 (31,1–36,4)	1,0 (0,95–1,03)	0,673
	Menos de superior	3.787	43,8 (41,7–45,9)	583	19,2 (16,4–22,3)	1,1 (1,12–1,17)	< 0,001
	Universidad o más	607	7,8 (6,2–9,8)	27	0,5 (0,3–0,8)	1,0 (0,98–1,07)	0,478
Inseguridad Alimentaria^b^							
	No	3.194	40,4 (38,3–42,6)	969	31,7 (28,6–34,9)	1,1 (1,05–1,13)	< 0,001
	Leve	3.282	37,6 (35,5–39,6)	1.236	35,7 (32,9–38,7)	1,0 (0,99–1,07)	0,234
	Moderada	1.437	13,4 (12,3–14,6)	690	18,3 (16,1–20,7)	0,9 (0,80–0,97)	0,004
	Severa	936	8,6 (7,5–9,9)	660	14,3 (11,9–17,0)	0,8 (0,65–0,90)	< 0,001
Disponibilidad de agua							
	Acueducto público	6.171	84,6 (82,3–86,4)	634	22,6 (19,3–26,2)	1,5 (1,42–1,53)	< 0,001
	Pozo o comunal	353	1,5 (1,04–2,03)	1.166	34,0 (29,7–38,7)	0,0 (0,02–0,10)	< 0,001
	Agua embotellada	1.311	10,0 (8,67–11,6)	161	4,1 (3,05–5,60)	1,1 (1,03–1,12)	0,020
	Otro	1.016	3,9 (2,98–5,15)	1.599	39,3 (34,4–44,3)	0,9 (0,84–0,91)	< 0,001
Alcantarillado							
	Si	6924	89,9 (88,0–91,5)	378	16,2 (12,5–20,6)	3,1 (2,96–3,35)	<0,001
	No	1.927	10,1 (8,5–12,0)	3.182	83,8 (79,4–87,5)	0,0 (0,03–0,05)	<0,001
Diarrea^c^							
	Si	1.057	29,0 (26,6–31,6)	428	38,0 (32,6–43,6)	1,2 (1,08–1,27)	< 0,001
	No	2.314	71,0 (68,4–73,4)	703	62,0 (56,4–67,4)	1,1 (1,06–1,16)	< 0,001
Infección respiratoria^c^							
	Si	2.634	79,8 (77,6–81,8)	873	77,6 (72,9–81,6)	1,0 (0,99–1,09)	0,155
	No	737	20,2 (18,2–22,4)	258	22,4 (18,4–27,1)	1,0 (0,88–1,06)	0,441

a RP: razón de prevalencia [urbano/rural].

b Con base la ELCSA.

c En los últimos quince días, por auto reporte.

Cuando el jefe del hogar tuvo menos de secundaria, los niños que habitaban en la zona rural presentaron menos déficit de peso para la talla que los que habitaban en la zona urbana, p = 0,013. Igual ocurrió en los niños sin aseguramiento o pertenecientes al régimen contributivo y en los que habitan en hogares con INSA leve y moderada (Tabla 2). Los niños que habitaban en hogares clasificados con INSA moderada tuvieron más exceso de peso para la talla que aquellos que habitaban en la zona urbana, RP = 3,8 (IC95% 1,7–8,8), p = 0,002. Lo mismo ocurrió en aquellos niños clasificados en la tercil más bajo del índice de riqueza y los que habitan en la región Atlántica (Tabla 3).

**Tabla 2:** Prevalencia de déficit en el indicador peso/talla (Z < −2) en niños colombianos entre cero y cuatro años por área geográfica. Colombia, ENSIN–2015.

Variable		Urbano (n = 8.735)		Rural (n = 3.521)		RPaj^a^ (IC95%)	p
		Prevalencia (IC95%)	p	Prevalencia (IC95%)	p		
Total		1,4 (1,13–1,83)		1,8 (1,29–2,57)	0,282	0,7 (0,44–1,10)	0,120
Sexo			0,753		0,766		
	Hombre	1,5 (1,12–2,00)		1,9 (1,13–3,25)		0,8 (0,40–165)	0,563
	Mujer	1,4 (0,94–2,04)		1,7 (1,07–2,76)		0,6 (0,31–1,10)	0,093
Grupo de edad (meses)			0,001		0,004		
	0–11	2,4 (1,64–3,47)		2,9 (1,31–6,50)		0,6 (0,25–1,37)	0,211
	12–23	1,7 (1,12–2,61)		2,7 (1,41–4,98)		0,5 (0,25–1,13)	0,099
	24–35	1,5 (0,75–3,19)		1,5 (0,60–3,70)		0,9 (0,33–2,34)	0,790
	36–48	0,5 (0,02–1,02)		1,9 (1,00–3,72)		4,7 (0,65–33,65)	0,124
	48–59	1,1 (0,67–1,86)		0,0 (0,00–0,34)		0,0 (0,00–0,32)	0,004
Retraso en talla (T/E)			0,272		0,798		
	No	1,5 (1,16–1,91)		1,8 (1,21–2,66)		0,7 (0,41–1,36)	0,139
	Si (Z < −2)	1,0 (0,50–1,98)		2,0 (1,06–3,65)		0,7 (0,28–1,91)	0,511
Escolaridad del jefe			0,984		0,058		
	Menos de primaria	1,5 (0,90–2,54)		2,7 (1,76–4,06)		1,0 (0,44–2,14)	0,944
	Menos de secundaria	1,2 (0,80–1,75)		0,9 (0,37–2,29)		0,3 (0,12–0,77)	0,013
	Menos de superior	1,8 (1,24–2,61)		1,3 (0,57–3,12)		0,6 (0,22–1,62)	0,303
	Universidad o más	0,6 (0,25–1,24)		no		nd	nd
Tipo de aseguramiento			0,009		< 0,001		
	Sin aseguramiento	1,1 (0,64–1,70)		0,2 (0,00–0,73)		0,1 (0,04–0,31)	< 0,001
	Subsidiado	1,7 (1,26–2,19)		2,2 (1,51–3,13)		0,8 (0,50–1,40)	0,490
	Contributivo/otro	4,1 (2,12–5,46)		1,5 (0,47–4,57)		0,2 (0,05–0,84)	0,029
Inseguridad Alimentaria^b^			0,114		0,132		
	No	1,2 (0,68–1,99)		1,7 (0,84–3,30)		1,1 (0,45–2,57)	0,872
	Leve	1,5 (1,09–2,10)		1,2 (0,69–2,12)		0,4 (0,19–0,97)	0,043
	Moderada	1,5 (0,84–2,67)		1,5 (0,72–3,08)		0,4 (0,14–0,92)	0,034
	Severa	2,3 (1,34–4,02)		4,1 (1,99–8,34)		0,8 (0,38–1,58)	0,476
Índice de riqueza			0,010		0,077		
	T_1_- los más pobres	2,8 (1,76–4,60)		2,2 (1,54–3,19)		0,7 (0,40–1,26)	0,239
	T_2_	1,6 (1,16–2,19)		0,6 (0,12–2,58)		0,4 (0,07–1,70)	0,190
	T_5_- los más ricos	1,1 (0,67–1,70)		no		nd	nd
Etnia			0,119		0,174		
	Mestizo	1,3 (1,01–1,76)		1,6 (1,13–2,31)		0,6 (0,36–1,05)	0,072
	Negro/Afro	2,5 (1,47–4,33)		1,1 (0,41–2,77)		0,3 (0,07–1,58)	0,152
	Indígena	1,0 (0,32–3,37)		3,6 (1,41–8,92)		1,5 (0,44–5,33)	0,473
Región			0,116		0,068		
	Central	1,1 (0,67–1,76)		2,4 (1,45–4,01)		0,7 (0,28–1,55)	0,318
	Atlántico	2,3 (1,69–3,03)		2,2 (1,05–4,59)		0,5 (0,25–1,05)	0,067
	Oriental	1,8 (0,79–3,85)		0,9 (0,50–1,65)		1,5 (0,63–3,34)	0,350
	Pacífico	1,2 (0,65–2,26)		1,8 (0,95–3,40)		1,0 (0,23–4,26)	0,994
	Bogotá	0,9 (0,41–1,99)		no		nd	nd
	Amazonía/Orinoquia	0,6 (0,31–1,15)		no		nd	nd

no: sin observaciones; nd: no disponible.

a RPaj: razón de prevalencia ajustada [rural/urbano] alcanzadas en un modelo binomial, con la prevalencia de déficit en el indicador P/T, Z < −2, como la variable dependiente y el área como la principal explicación. El ajuste se realizó por las siguientes covariables: el sexo, la edad, el retraso en talla T/E, Z < −2, el nivel de escolaridad del jefe del hogar, el tipo de aseguramiento, el nivel de inseguridad alimentaria del hogar, el índice de riqueza, la etnia y la región.

b Con base la ELCSA.

**Tabla 3:** Prevalencia de exceso en el indicador peso/talla (Z > 2) en niños colombianos entre cero y cuatro años por área geográfica. Colombia, ENSIN–2015.

Variable		Urbano (n = 8.735)		Rural (n = 3.521)		RPaj^a^ (IC95%)	p
		Prevalencia (IC95%)	p	Prevalencia (IC95%)	p		
Total		5,7 (4,98–6,52)		5,5 (4,20–7,12)	0,672	1,3 (0,83–2,01)	0,244
Sexo			0,013		0,093		
	Hombre	6,6 (5,52–7,93)		6,3 (4,64–8,54)		1,3 (0,82–2,11)	0,251
	Mujer	4,7 (3,90–5,73)		4,6 (3,24–6,48)		1,3 (0,74–2,27)	0,360
Grupo de edad (meses)			0,182		0,001		
	0–11	6,2 (4,63–8,32)		6,7 (4,56–9,80)		1,2 (0,73–1,99)	0,460
	12–23	6,2 (4,76–8,01)		7,9 (4,72–12,9)		2,0 (0,91–4,51)	0,081
	24–35	6,5 (4,75–8,85)		5,3 (3,40–8,14)		0,8 (0,41–1,74)	0,639
	36–48	4,1 (3,07–5,49)		5,4 (3,02–9,47)		2,4 (0,80–7,00)	0,119
	48–59	5,5 (4,09–7,32)		2,0 (1,08–3,81)		0,5 (0,21–1,34)	0,178
Retraso en talla (T/E)			0,132		0,239		
	No	5,5 (4,77–6,35)		5,2 (4,10–6,53)		1,2 (0,85–1,82)	0,247
	Si (Z < −2)	7,7 (5,06–11,5)		7,1 (3,86–12,7)		1,8 (0,57–5,78)	0,302
Escolaridad del jefe			0,351		0,995		
	Menos de primaria	5,9 (4,24–8,19)		5,2 (3,13–8,65)		1,4 (0,31–6,32)	0,656
	Menos de secundaria	5,1 (4,03–6,42)		5,8 (4,23–7,90)		1,4 (0,92–2,25)	0,107
	Menos de superior	5,8 (4,81–7,00)		5,3 (3,49–7,86)		1,1 (0,68–1,89)	0,616
	Universidad o más	7,9 (4,56–13,4)		no		nd	nd
Tipo de aseguramiento			0,003		0,754		
	Sin aseguramiento	7,0 (5,81–8,43)		6,3 (3,46–11,1)		0,8 (0,45–1,52)	0,562
	Subsidiado	4,3 (3,52–5,17)		5,3 (3,81–7,23)		1,7 (0,85–3,43)	0,133
	Contributivo/otro	5,5 (3,00–9,78)		6,2 (3,58–10,4)		2,0 (0,46–8,73)	0,342
Inseguridad alimentaria^b^			0,024		0,641		
	No	7,2 (5,86–8,88)		5,0 (3,69–6,81)		0,7 (0,48–1,18)	0,210
	Leve	4,3 (3,46–5,38)		6,2 (4,50–8,56)		1,5 (0,96–2,36)	0,077
	Moderada	6,1 (4,39–8,48)		6,6 (2,54–15,9)		3,8 (1,67–8,80)	0,002
	Severa	4,0 (2,43–6,43)		3,2 (2,01–5,23)		1,2 (0,52–2,82)	0,649
Índice de riqueza			<0,001		0,677		
	T_1_- los más pobres	2,6 (1,69–3,85)		5,2 (4,23–6,33)		2,0 (1,30–3,19)	0,002
	T_2_	5,1 (4,17–6,25)		6,8 (3,01–14,6)		1,2 (0,50–3,04)	0,649
	T_5_- los más ricos	6,7 (5,57–8,07)		1,5 (0,20–9,97		0,2 (0,03–1,38)	0,102
Etnia			0,097		0,359		
	Mestizo	5,8 (5,02–6,71)		5,7 (4,12–7,78)		1,3 (0,75–2,15)	0,367
	Negro/Afro	4,9 (3,20–7,48)		5,1 (3,19–8,08)		1,9 (0,75–4,72)	0,162
	Indígena	2,4 (1,42–4,19)		4,2 (2,41–7,25)		2,3 (0,98–5,40)	0,056
Región			0,704		0,121		
	Central	6,5 (5,04–8,39)		7,5 (4,00–13,7)		1,8 (0,54–6,19)	0,306
	Atlántico	5,0 (4,14–6,05)		4,4 (3,09–6,12)		2,4 (1,43–3,95)	0,003
	Oriental	5,8 (3,91–8,64)		6,2 (3,91–9,71)		0,7 (0,28–1,53)	0,305
	Pacífico	5,3 (3,63–7,81)		4,8 (3,36–6,69)		1,2 (0,52–2,60)	0,680
	Bogotá	5,7 (3,95–8,27)		no		nd	nd
	Amazonía/Orinoquia	5,9 (4,59–7,48)		0,1 (0,00–1,97)		7,6 (1,29–44,86)	0,011

no: sin observaciones; nd: no disponible.

a RPaj: razón de prevalencia ajustada [rural/urbano] alcanzadas en un modelo binomial, con la prevalencia de déficit en el indicador P/T, Z < −2, como la variable dependiente y el área como la principal explicación. El ajuste se realizó por las siguientes covariables: el sexo, la edad, el retraso en talla T/E, Z < −2, el nivel de escolaridad del jefe del hogar, el tipo de aseguramiento, el nivel de inseguridad alimentaria del hogar, el índice de riqueza, la etnia y la región.

b Con base la ELCSA.

El retraso en talla fue mayor en la zona rural, RP = 1,24 (IC95% 1,00–1,53), p = 0,050; lo mismo ocurrió al comparar las prevalencias de retraso en T/E en la zona rural para algunas de las categorías de las covariables; los menores de un año, los niños con exceso de peso para la talla cuyos padres tienen menor escolaridad, aquellos que viven en hogares con INSA moderada y en la región central, tienen más retraso en talla si habitan en la zona rural (Tabla 4).

**Tabla 4:** Prevalencia de déficit en el indicador talla/edad (Z > -2) en niños colombianos entre cero y cuatro años por área geográfica. Colombia, ENSIN-2015.

Variable		Urbano (n = 8.735)		Rural (n = 3.521)		RPaj^a^ (IC95%)	p
		Prevalencia (IC95%)	p	Prevalencia (IC95%)	p		
Total		9,0 (7,96–10,2)		15,4 (13,7–17,3)	0,004	1,2 (1,00–1,53)	0,050
Sexo			0,041		0,033		
	Hombre	10,0 (8,67–11,6)		17,4 (15,1–20,0)		1,2 (0,97–1,59)	0,087
	Mujer	7,9 (6,52–9,61)		13,3 (10,8–16,3)		1,2 (0,85–1,84)	0,246
Grupo de edad (meses)			0,102		0,010		
	0–11	5,4 (3,14–9,17)		8,1 (5,32–12,1)		2,2 (1,20–3,89)	0,011
	12–23	9,4 (7,31–12,1)		16,2 (12,3–21,2)		1,4 (0,81–2,52)	0,220
	24–35	12,3 (10,0–14,9)		21,2 (17,3–25,7)		0,9 (0,68–1,26)	0,615
	36–48	9,2 (7,43–11,3)		16,7 (13,2–21,0)		0,3 (0,83–2,15)	0,227
	48–59	8,8 (7,04–10,9)		14,2 (11,1–18,1)		1,2 (0,80–1,72)	0,401
Estado nutrición (P/T)			< 0,001		< 0,001		
	DNT aguda (Z < −2)	6,2 (3,14–12,0)		16,7 (8,76–2,94)		1,6 (0,40–6,20)	0,491
	Normal	8,9 (7,80–10,1)		15,1 (13,3–17,1)		1,2 (0,97–1,51)	0,093
	Exceso (Z > 2)	12,2 (8,04–18,0)		20,0 (12,5–30,4)		1,7 (1,30–2,33)	< 0,001
Escolaridad del jefe			< 0,001		0,020		
	Menos de primaria	10,5 (8,14–13,4)		18,3 (15,7–21,2)		1,7 (1,21–2,50)	0,003
	Menos de secundaria	11,4 (9,33–13,9)		13,0 (10,4–16,2)		0,8 (0,58–1,20)	0,326
	Menos de superior	7,3 (6,15–8,72)		12,4 (8,02–18,6)		1,5 (1,03–2,10)	0,034
	Universidad o más	4,8 (2,76–8,09)		11,8 (2,22–44,0)		2,3 (0,87–6,00)	0,089
Tipo de aseguramiento			0,011		0,639		
	Sin aseguramiento	7,3 (5,78–9,16)		13,3 (9,04–19,2)		1,8 (1,07–2,94)	0,027
	Subsidiado	10,5 (9,17–11,9)		16,0 (14,1–18,1)		1,1 (0,90–1,42)	0,301
	Contributivo/otro	9,0 (5,97–13,5)		12,9 (8,48–19,2)		nd	nd
Inseguridad alimentaria^b^			0,081		< 0,001		
	No	8,6 (6,76–10,8)		10,2 (7,96–13,0)		0,8 (0,53–1,19)	0,252
	Leve	8,1 (6,72–9,84)		13,1 (10,3–16,5)		1,1 (0,79–1,65)	0,470
	Moderada	11,0 (8,92–13,5)		18,8 (14,7–23,6)		1,7 (1,09–2,59)	0,019
	Severa	11,8 (8,72–15,8)		28,4 (24,3–33,0)		1,5 (1,01–2,26)	0,044
Índice de riqueza			0,001		0,089		
	T_1_- los más pobres	13,5 (10,8–16,8)		16,3 (14,5–18,3)		1,2 (0,91–1,52)	0,203
	T_2_	10,0 (8,61–11,6)		12,5 (7,99–19,0)		1,3 (0,82–2,03)	0,264
	T_5_- los más ricos	7,5 (5,96–9,32)		11,1 (1,58–49,1)		nd	nd
Etnia			0,720		< 0,001		
	Mestizo	9,0 (7,88–10,3)		12,8 (11,0–14,9)		1,0 (0,79–1,35)	0,796
	Negro/Afro	5,7 (3,78–8,60)		10,7 (7,97–14,3)		1,4 (0,68–3,00)	0,319
	Indígena	17,8 (11,9–25,8)		34,0 (29,1–39,3)		1,3 (0,75–2,34)	0,312
Región			0,008		0,337		
	Central	7,1 (5,53–9,15)		15,5 (12,1–19,5)		2,0 (1,02–3,76)	0,043
	Atlántico	8,7 (7,38–10,3)		19,4 (16,7–22,4)		1,0 (0,73–1,31)	0,863
	Oriental	8,3 (5,96–11,5)		11,6 (8,93–14,8)		0,9 (0,55–1,65)	0,840
	Pacífico	7,5 (5,55–10,1)		13,9 (9,77–19,4)		1,4 (0,71–2,70)	0,303
	Bogotá	13,1 (9,43–17,9)		no		nd	nd
	Amazonía/Orinoquia	10,4 (8,93–12,1)		16,2 (6,23–36,1)		nd	nd

no: sin observaciones; nd: no disponible.

a RP*aj*: razón de prevalencia ajustada [rural/urbano] alcanzadas en un modelo binomial, con la prevalencia de déficit en el indicador P/T, Z < −2, como la variable dependiente y el área como la principal explicación. El ajuste se realizó por las siguientes covariables: el sexo, la edad, el estado de nutrición P/T, el nivel de escolaridad del jefe del hogar, el tipo de aseguramiento, el nivel de inseguridad alimentaria del hogar, el índice de riqueza, la etnia y la región.

b Con base la ELCSA.

## DISCUSIÓN

Con base en la ENSIN-2015, se estimó en menores de cinco años la prevalencia de déficit y exceso en el indicador P/T y el déficit en el indicador T/E. La prevalencia de déficit para la talla, pasó de 13,2% en 2010^7^ a 10,8% en 2015. Sin embargo, el déficit y exceso en el indicador P/T aumentaron, la desnutrición aguda pasó de 0,9% en 2010^7^ a 1,6% en 2015 y la de exceso en el indicador P/T de 5,2% en 2010^7^ a 5,6% en 2015. Además, se estableció que no hay diferencias estadísticamente significativas por zona geográfica en las prevalencias de déficit y exceso en el indicador P/T, pero sí en el indicador T/E.

El indicador T/E en Colombia es menor que el promedio reportado por FAO para 2018 en los países asiáticos, 22,7%, o africanos, 30,0% y mayor que el promedio en los países de ingresos altos, 3,0% y que el promedio en América Latina, 9,0%. El exceso de peso (P/T) en Colombia es mayor que el promedio en los países asiáticos, 5,2%, africanos, 4,9% y menor que en los de América Latina, 7,5% y los países de ingresos altos como Estados Unidos, 7,2%^1^.

Según el Banco Mundial y el Fondo de las Naciones Unidas para la Infancia (UNICEF), la prevalencia de déficit en el indicador T/E viene disminuyendo en todos los países de la región clasificados como de ingreso medio alto como Colombia^27^. Las últimas cifras disponibles para menores de cinco años y derivadas de encuestas nacionales fluctúan levemente alrededor de la aquí reportada, para Argentina (2005) 8,2%, Costa Rita (2009) 5,6%, Cuba (2000) 7,0%, República Dominicana (2013) 7,1%, Ecuador (2014) 23,9%, Jamaica (2014) 6,2%, México (2016) 12,4%, Panamá (2008) 19,1%, Paraguay (2016) 5,6%, Perú (2016) 13,1%, Venezuela (2009) 13,4%^27^.

Complementariamente, el exceso de peso en el mismo indicador es levemente mayor al aquí reportado en los países de ingreso medio alto: en Argentina (2005) 9,9%, en Costa Rita (2009) 8,1%, en República Dominicana (2013) 7,6%, en Ecuador (2014) 8,0%, en Jamaica 8,5%, en México (2016) 5,2%, en Panamá (1997) 6,2%, en Paraguay (2016) 12,4%, en Perú (2016) 7,2% y en Venezuela (2009) 6,4% (46). La prevalencia de déficit en el indicador P/T está por debajo del 2,5% teórico en estos países. En Estados Unidos (2012), un país de ingreso alto, el déficit en T/E es de 2,1% y el de exceso en P/T de 6,0%^27^.

La clasificación de urbano y rural en la ENSIN-2015 es basada en la concentración de la población que desconoce el concepto de territorio y las siete dimensiones constitutivas del mismo^9^^,^^28^. Sin embargo, el concepto de rural, como se ha concebido tradicionalmente, engloba a las relaciones de los sujetos que allí habitan y que son predominantemente con el agro^2^^,^^28^. La pobreza u otras variables –aquí el estado de nutrición-, alcanzando valores similares en lo rural y urbano son teóricamente diferentes, dado que resultan de procesos estructuralmente disimiles en la zona geográfica. Por ejemplo, a) los bienes y los servicios son más costosos en lo urbano que en lo rural, b) la supervivencia urbana exige de bienes y servicios que en el campo no son necesarios, c) el dinero es más importante en la zona urbana que en la rural, d) los hogares urbanos son más vulnerables que los rurales, e) la posición del jefe del hogar en lo urbano es determinante del mercado de trabajo, en lo rural no, f) el desarrollo impacta a lo urbano, poco o nada a lo rural y g) el acceso a alimentos en lo urbano se hace casi exclusivamente a través de dinero, en lo rural no. Además, establecimos que la escolaridad del jefe y la INSA del hogar, y otras variables de infraestructura básica como la disponibilidad de agua y alcantarillado, son desiguales por la zona geográfica (Tabla 1).

Respecto al déficit en el indicador P/T, la escolaridad del jefe del hogar – tener menos de secundaria, el tipo de aseguramiento – sin aseguramiento y régimen contributivo, y la INSA – leve o moderada, se asociaron de manera coherente con lo ya advertido. Las RP en las categorías de estas variables muestran que el déficit agudo es menor en la zona rural. Cuando se ajustan las prevalencias, a pesar de menores capacidades individuales como la educación^29^, o de institucionalidad como el aseguramiento en el sistema de salud, existe menor déficit en P/T asociado a la INSA leve y moderada en lo rural que en lo urbano (Tabla 2). Este hallazgo controvierte lo que hasta ahora conocemos para el caso colombiano e invita a repensar las desigualdades por zona geográfica. Las dinámicas actuales en las variables estudiadas se convierten en potenciales confusoras de la prevalencia de déficit en P/T.

Los más pobres según el índice de riqueza, los hogares con INSA moderada y la región del Atlántico de la que hacen parte departamentos pobres como la Guajira, presentaron más exceso de peso en lo rural que en lo urbano (Tabla 3). El exceso asociado a mayor pobreza ya se había observado cuando se declaró la transición nutricional para América Latina^14^, en Colombia con datos de la ENSIN-2010^4^ y en niños colombianos^30^.

El déficit en T/E corrobora que las menores capacidades determinantes del desarrollo social (la escolaridad del jefe del hogar), que a su vez llevan a menor acceso a los alimentos (INSA), sumadas a un entorno con desigualdades institucionales (menor aseguramiento en salud), se traducen a más temprana edad (en los menores de un año) en retraso en T/E. En lo biológico, el círculo vicioso de más obesidad en los niños rurales y además, con retraso en talla, ilustra lo ya descrito en la década de los 60´s^31^.

La ruralidad colombiana presenta desigualdades crónicas, que limitan en alto grado el desarrollo humano, social, estructural y económico; sus efectos son visibles a nivel de los individuos y los colectivos sociales. Este estado crónico de subdesarrollo relativo al urbano aún se evidencia en el estado de nutrición, y aunque la pobreza disminuye, no lo hace la desigualdad. Las formas de desarrollo y subdesarrollo cambian y a pesar de esto, el estado de nutrición sigue siendo sensible a éstos. El mayor aporte de este estudio no es sobre si existen desigualdades entre lo rural y lo urbano, pues hace más de 70 años para el caso colombiano son innegables. La evidencia sigue vigente en todos los países de la región, incluido Brasil^32^ y otros de ingresos bajos^33^. El mayor aporte es que estableció de un conjunto de variables que expresan aristas del desarrollo en Colombia, dos relevantes que hoy influyen más sobre el estado de nutrición. Incrementar las capacidades individuales – a través de la educación como estrategia de mediano y largo plazo – y garantizar el acceso a los alimentos – con medidas de corto, mediano y largo plazo – son las variables claves para impactar positivamente el estado de nutrición de los menores de cinco años y la población en general. Lo anterior corrobora la intencionalidad del estado colombiano^2^^,^^3^^,^^28^ y la ONU^17^.

Los datos analizados provienen de una encuesta nacional y a través de análisis multivariado, se limpió el potencial efecto confusor de variables que tradicionalmente se han ligado al estado de nutrición en menores de cinco años. Los métodos usados para derivar la clasificación tanto del estado de nutrición como de la zona geográfica son aceptados universalmente y siguen siendo sensibles a la desigualdad en las condiciones actuales y nivel de desarrollo alcanzado en Colombia. La principal limitación es que proviene de datos de corte transversal. Sin embargo, la coherencia con el conocimiento alcanzado al menos en los últimos 100 años, le confieren validez externa al estudio.

La prevalencia en los indicadores del estado de nutrición sigue las tendencias regionales y mundiales. No hay diferencias por zona geográfica en las prevalencias de déficit y exceso en el indicador P/T, pero sí en el indicador T/E. El indicador T/E es el mejor para establecer el nivel de desarrollo geográfico y las brechas urbano *versus* rural. Algunas expresiones del desarrollo económico y social se relacionaron con el estado de nutrición, siendo la escolaridad del jefe y la inseguridad alimentaria del hogar las más importantes. La transición nutricional en Colombia está vigente y es dinámica. Estos hallazgos permitirán el diseño y ajuste de las políticas públicas relacionadas para logar costo-eficiencia.
